# Sex differences in the reactivity of gastric myoelectrical activity and heart rate variability as putative psychophysiological markers in human pain research

**DOI:** 10.3389/fnins.2024.1502752

**Published:** 2024-12-11

**Authors:** Rossitza Draganova, Genisius Hartanto, Robert Jan Pawlik, Jana Luisa Aulenkamp, Sigrid Elsenbruch

**Affiliations:** ^1^Department of Neurology, Center for Translational Neuro-and Behavioral Sciences (C-TNBS), University Hospital Essen, University of Duisburg-Essen, Essen, Germany; ^2^Department of Medical Psychology and Medical Sociology, Ruhr University Bochum, Bochum, Germany; ^3^Department of Anesthesiology and Intensive Care Medicine, University Hospital Essen, University of Duisburg-Essen, Essen, Germany

**Keywords:** sex differences, visceral pain, gut-brain axis, electrogastrography, heart rate variability, psychophysiology, autonomic nervous system, stress

## Abstract

**Background:**

This study explored the potential of electrogastrography (EGG) and heart rate variability (HRV) as psychophysiological markers in experimental pain research related to the gut-brain axis. We investigated responses to the experience of pain from the visceral (rectal distension) and somatic (cutaneous heat) pain modalities, with a focus on elucidating sex differences in EGG and HRV responses.

**Methods:**

In a sample of healthy volunteers (29 males, 43 females), EGG and ECG data were collected during a baseline and a pain phase. Data were analyzed for changes in gastric myoelectrical activity and cardiac autonomic regulation, with special attention to sex-specific patterns and correlations with perceptual responses to visceral and somatic pain stimuli, assessed by visual analogue scale ratings.

**Results:**

Acute pain induced significant instability in EGG slow-wave frequency and amplitude, increased tachygastria, and decreased normogastric spectral power, without evidence of sex differences. HRV analyses revealed increases in SDNN, RMSSD, and pNN50 during pain, indicating sympathovagal regulation changes. While there were no significant sex differences in EGG responses, only female participants exhibited significant correlations between visceral pain unpleasantness and EGG alterations. HRV measures, particularly time-domain parameters, showed sex differences, independent of pain-induced autonomic reactivity.

**Conclusion:**

The experience of pain in the lower abdominal region may induce impaired gastric motility. EGG and HRV are sensitive to acute pain and offer insight into pain mechanisms along the gut-brain axis. While EGG responses were consistent across sexes, HRV revealed sex-specific differences, suggesting that autonomic regulation and gastric motility may be modulated differently by pain and psychosocial factors. Further research in patients with chronic visceral pain is warranted.

## 1 Introduction

Disorders of gut-brain interactions (DGBI), such as irritable bowel syndrome (IBS) and functional dyspepsia (FD), are highly prevalent, especially in women. Visceral pain, a key IBS symptom with significant socioeconomic and personal impact, is challenging to treat, highlighting the need for research on sex differences in pain mechanisms to improve treatment (Labrenz et al., 2020; Keogh, 2022). One unresolved challenge is that IBS symptoms are often not limited to the lower gastrointestinal (GI) tract but can include fluctuating pain or discomfort in the upper and lower abdomen, nausea, postprandial fullness, and other related GI symptoms. Overlap with other GI or somatic conditions is common (Lackner et al., 2013; Parkman et al., 1996), likely due to shared pathophysiological mechanisms, such as autonomic nervous system (ANS) dysfunction, in line with the broad role of stress in the modulation of both the intestinal barrier and the brain-gut-microbiota axis (Labanski et al., 2020). The ANS plays a recognized role in interoception, visceral pain, and DGBI (Craig, 2002; Meissner, 2014), and acute somatic pain also reportedly triggers autonomic responses (Forte et al., 2022), with possible sex differences in the relationships between parasympathetic activity and pain modulation (Nahman-Averbuch et al., 2016a).

One method that could shed light on ANS and pain modulation is electrogastrography (EGG). EGG is a non-invasive, well-established tool for studying symptoms with autonomic nervous system involvement, especially nausea, vomiting, and postprandial fullness. It has been a widely used research tool in both animal and human neurogastroenterology (Yin and Chen, 2013) and psychophysiology (Wolpert et al., 2020), but has rarely been applied in the context of pain research. EGG measures stomach myoelectric activity using cutaneous electrodes on the abdomen (Chen et al., 1999; Parkman et al., 2003; Yin and Chen, 2013). This activity, generated by Interstitial Cells of Cajal (ICCs), forms the gastric rhythm or “slow waves” (Wolpert et al., 2020). Slow waves control smooth muscle contractions with input from enteric motor neurons, vagal efferents, and smooth muscle cells. EGG captures (i) slow-wave rhythm and/or (ii) myoelectric activity with muscle contractions. Dysrhythmias, like tachygastric (abnormally fast) and bradygastric (abnormally slow) activity, are linked to nausea (Koch, 2014) and other upper GI symptoms common in DGBI patients (Koch, 2003; Riezzo et al., 2013). EGG has also proven fruitful in basic neurogastroenterology research to elucidate the effects of psychosocial factors and sex differences, such as placebo/nocebo effects on nausea or motion sickness (Levine et al., 2006; Meissner et al., 2020) and to elucidate mechanisms underlying disgust (Meissner et al., 2011). However, its use in biobehavioral pain research is limited, with only one animal study that tested the effects of rectal-distension-induced pain (Abo et al., 2000), and no known human studies on stomach myoelectric changes in response to acute pain.

Synchronization between ICCs, enteric neurons, smooth muscle, and the ANS is essential for normal gastric function (Koch, 2014). Combining EGG with specific ANS markers, like heart rate variability (HRV), has therefore been implemented in studies (e.g., Meissner, 2009). HRV, a marker of cardiac autonomic regulation, has been linked to reactivity to experimental pain (see Forte et al., 2022). However, HRV responses to pain vary due to factors like pain model, sex, and subjective experience. Most studies used somatic stimuli, with only two involving visceral stimulation (esophageal balloon distention, see Paine et al., 2009a, 2009b). Research on autonomic responses to clinically relevant pain remains incomplete, and no studies have explored EGG and HRV changes during acute visceral and somatic pain in healthy humans.

In this analysis, we aimed to determine whether EGG and HRV outcomes can serve as reliable psychophysiological markers in experimental pain research at the intersection of the gut-brain axis and pain studies. Drawing from a larger investigation on nocebo effects in pain (Aulenkamp et al., 2023), we collected EGG and electrocardiogram (ECG) data from a relatively large sample of healthy male and female volunteers during a baseline (pain-free) and a pain phase. The pain phase, consistent with our previous studies (Koenen et al., 2017, 2021; Lanters et al., 2024), involved individually calibrated visceral and somatic pain stimuli (rectal distensions and thermal cutaneous stimuli). These stimuli target interoceptive and exteroceptive nociceptive pathways, simulating the range of symptoms often experienced by patients with chronic abdominal pain or somatic symptom disorders. Given the limited understanding of sex differences, and prior findings of sex-specific responses to heat pain (Nahman-Averbuch et al., 2016a) and placebo effects on gastric myoelectric activity exclusively in females (Meissner et al., 2020), we also investigated sex differences in EGG and HRV responses to pain stimuli. This investigation would subsequently unveil the potential of both neurophysiological measures as valuable tools for capturing complementary facets of pain processing, particularly regarding sex differences, and thereby expand future understanding of pain mechanisms in DGBI.

## 2 Methods

### 2.1 Participants

Healthy adult volunteers within an age range of 18–45 years and a body mass index (BMI) >18 and < 30 kg/m^2^ were recruited for a comprehensive experimental study on cognitive pain modulation. The study was primarily designed to elucidate nocebo effects in the visceral and somatic pain modalities and comprised two experimental study days scheduled 7 days apart. For the purposes herein, we analyzed psychophysiological data acquired on study day 2 in a subset of participants, as detailed below. The study was approved by the local Ethics Committee of the University Hospital Essen (protocol number 19-8897-BO), preregistered in the German Clinical Trials Register (DRKS: DRKS00024410), and is described in detail in a published study protocol (Aulenkamp et al., 2023). All volunteers signed consent and received financial compensation for their participation.

The standardized screening process and exclusion criteria for pain studies in healthy volunteers followed our established procedures. In brief, any medical condition, current medication use, gastrointestinal complaints, anal tissue damage, or current symptoms of anxiety or depression led to exclusion. For the present analysis, data from a total of 74 healthy volunteers were available for analyses of EGG and HRV (see Table 1 for sample characteristics). Due to artifacts, data from *N* = 2 participants were excluded from EGG analyses, and another *N* = 2 were excluded from HRV analyses.

**Table 1:** Sample characterization and pain perception.

Demographics	Mean ± SD			Independence *T*-test between groups (two-sided *p*-value)
	Total sample (*N* = 74)	Male (*N* = 29)	Female (*N* = 45)	
Age, years	24.89 ± 3.3	25.93 ± 3.49	24.22 ± 3.01	0.035
BMI	22.89 ± 2.76	23.9 ± 2.51	22.24 ± 2.74	0.009
Heat pain intensity rating	42.79 ± 20.5	44.82 ± 20.52	41.47 ± 20.61	0.497
Visceral pain intensity rating	46.43 ± 21.78	42.36 ± 20.38	49.07 ± 23.47	0.189
Heat pain unpleasantness rating	39.18 ± 22.2	36.51 ± 21.32	40.9 ± 22.82	0.404
Visceral pain unpleasantness rating	59.7 ± 21.74	54.55 ± 24.24	63.03 ± 19.53	0.12

Data are shown as mean ± standard deviation (SD). All ratings were acquired with visual analogue scales (VAS, 0–100 mm), completed after each pain stimulus and averaged for the Pain phase. *p*-values are results of independent-samples t-tests between male and female participants.

### 2.2 Study design and experimental procedures

The full study comprised two experimental study days with repeated delivery of acute visceral and somatic pain stimuli. On study day 1, pain stimuli were delivered with group-specific instruction and/or conditioning procedures. We herein analyzed pooled data collected on study day 2, accomplished 1 week after study day 1, from the subsets of participants with complete psychophysiological recordings. Importantly, all participants were treated the same on study day 2, and received neither treatment nor any specific instruction, allowing us to pool the data and explore responses in a pain exposure phase versus a control phase in healthy male and female participants. Specifically, an initial baseline phase without any painful stimuli (NoPain condition) was accomplished, which was followed by a pain exposure phase (Pain condition). Herein, a series of phasic painful visceral (rectal distension) and somatic (cutaneous thermal) pain stimuli were applied, using predetermined individually calibrated stimulation intensities, as detailed below. During each condition, continuous EGG and ECG recordings were acquired using cutaneous electrodes. The whole duration of the recordings lasted 10 min in the NoPain condition and 16 min in the Pain condition. Pain stimuli were rated with respect to perceived pain intensity and pain unpleasantness on visual analogue scales (VAS), used herein to verify suitable pain perception (manipulation check) as well as for correlational analyses.

### 2.3 Pain stimuli

A series of acute visceral and somatic pain stimuli was applied during the pain exposure phase comprising a total of 12 stimuli (6 visceral, 6 somatic). Phases were identical concerning the pseudorandomized order of stimuli, with all pain stimulus durations 20s each. For visceral pain, we applied pressure-controlled rectal distensions with a barostat system (Distender Series II™, 1,300 mL Single Balloon Barostat, G&J Electronics, Toronto, ON, Canada). Somatic pain stimuli were delivered via a thermal device (PATHWAY model CHEPS; Medoc Ltd. Advanced Medical Systems, Ramat Yishai, Israel), with the thermode positioned on the lower part of the abdomen. Stimulus intensities (i.e., pressure and temperature, respectively) were determined on study day 1, following an established multi-step procedure consisting of assessment of pain thresholds for each stimulus modality, calibration to an intensity achieving a moderately painful experience, matching of somatic to visceral perceptual intensity, and a brief pain habituation phase. Perceptual target intensity was 50 mm as perceived pain intensity on VAS with the endpoints 0 mm = “not painful at all” to 100 mm = “extremely painful.” In the matching procedure, the visceral and somatic stimuli were presented simultaneously and adjusted (decreased/increased) until participants rated both stimuli as equally painful. As established in our previous studies using the same procedure in several independent samples (Lanters et al., 2024; Koenen et al., 2021; Koenen et al., 2017), this reliably identifies individual stimulus identities (i.e., rectal distension pressure and temperature, respectively), effectively inducing suitable pain ratings with respect to largely comparable perceived intensity for visceral and somatic pain stimuli (see Table 1). As in previous work, unpleasantness ratings were higher for visceral than for somatic pain, without evidence of sex differences (Table 1).

### 2.4 EGG and ECG data acquisition

EGG and ECG were recorded with amplifiers of BIOPAC Systems MP160 with AcqKnowledge® 5.0.1. Software. Because of the very low-frequency characteristic of the EGG signal, the amplifier was set to low-pass filter (LP) at 1 Hz, high-pass filter (HP) at 0.005 Hz, and Gain 2000. The signal was digitized at a rate of 15.625 Hz. The recording of the ECG signal was set by the amplifier into the frequency range between 1 Hz and 35 Hz with a sampling rate of 2000 Hz.

Participants were instructed to abstain from physical exercise on the study day, and to omit food and drink (other than water) within 90 min of the scheduled arrival to the laboratory. They were also instructed not to apply body lotion on the abdomen given application of electrodes on the skin. All measurements were performed in a quiet room with participants placed in a supine position on a bed with a slightly raised upper body, maintaining comfort and a stable position during the recordings while allowing volunteers to perform ratings with a handheld device while viewing instructions on a monitor. Adhesive Ag/AgCl electrocardiographic electrodes were employed allowing a reliable acquisition of the EGG and ECG signals. As recommended by Yin and Chen (2013), the skin was beforehand abraded using a sandy skin-preparation jelly, and then a thin layer of electrode jelly was applied for a couple of minutes allowing it to penetrate the skin. Before placing the electrodes, the excessive jelly was completely wiped off.

**Figure 1:**
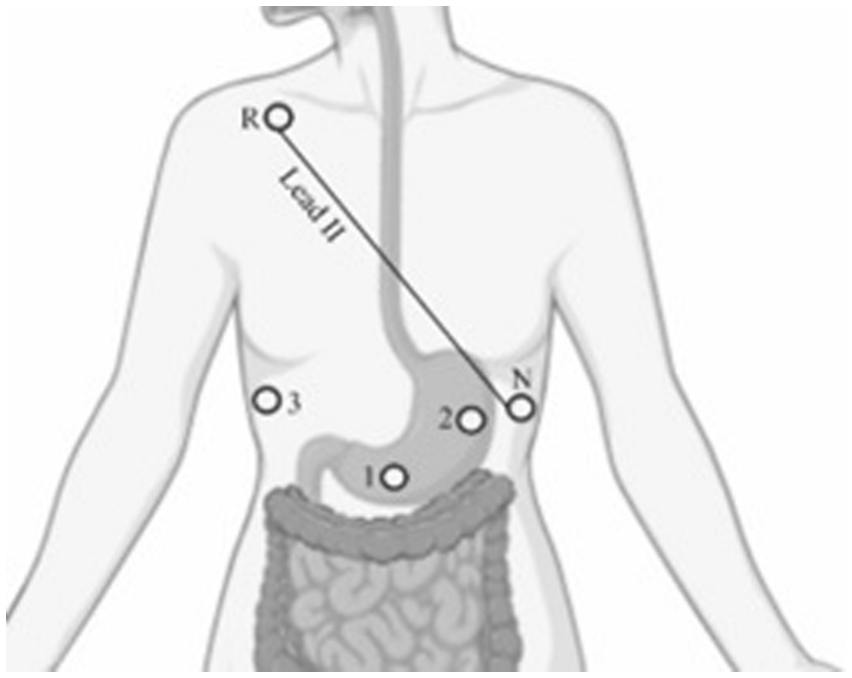
Illustration of EGG and ECG electrode positions. This figure was created using BioRender (www.biorender.com).

Regarding EGG electrode positioning, there exist several methods defining electrode positions for EGG recording dependent on the measurement goals (multichannel monopolar and bipolar). We herein accomplished bipolar recording using two active electrodes, placed on the curvatures between the fundus and the antrum in line with other research in humans (Chen et al., 1999; Meissner, 2009; Parkman et al., 2003; Yin and Chen, 2013). As illustrated in Figure 1, one active electrode (1) was placed at the lower 1/3 part of the line between the xiphoid and umbilicus. The second active electrode (2) was placed approximately 5 cm to the left of the first active electrode and around 30° cephalad, at least 2 cm below the rib cage in the midclavicular line. These electrode positions can vary depending on the body size of the subject, considering the reference position of the stomach anatomy. A reference electrode (3) was placed at a site distant from the active electrodes such as the right midclavicular line.

For ECG recording, an electrode used as a positive pole was situated at the lower left rib (N) on the Lead II bipolar axis (Einthoven schema, see Figure 1). The negative pole was placed at the upper right (R) just under the clavicle. Bipolar Lead II was chosen for recording because of the maximal current representation of the QRS-complex according to Einthoven’s law (Lead II = Lead I + Lead III).

### 2.5 Data analysis

#### 2.5.1 Electrogastrogram (EGG)

Given that the EGG has a frequency range between 0.0083 Hz and 0.2 Hz, during preprocessing the EGG signal was bandpass filtered (IIF filter) in BIOPAC Acqknowledge Software between 0.02 Hz and 0.15 Hz, following recommendations in the field (Parkman et al., 2003). This corresponds to a range of 1.2 to 9 cycles per minute (cpm, Hz/60). Thereafter, the digitized signal from Acqknowledge Software was transferred to a MAT file and further analyzed by custom-made MATLAB code. Data were then analyzed by the fast Fourier Transform (FFT) resulting in Power Spectral Density (PSD). For correct calculation of the FFT, the recorded EGG in both conditions was separated into 2-min segments (2048 samples) with an overlap of 1-min (1,024 samples), and for each 2-min segment, the PSD Running Spectrum was calculated. By plotting each 2-min EGG signal segment and its RS, a visual inspection was performed. Only artifact-free segments were accepted into further analyses. Given that harmonic frequencies due to the FFT are sometimes present, if a harmonic frequency corresponding to the dominant frequency was identified and was interfering within the tachygastric frequency range, tachygastric power of the particular segment was not included in further calculation. Following EGG studies in the field, (e.g., Meissner, 2009; Parkman et al., 2003; Yin and Chen, 2013), several quantitative parameters were analyzed: Based on the running spectra, these comprised the instability coefficients (IC) of the dominant frequency (IC_F) and amplitude (IC_A), respectively, and the normo-to-tachy ratio (NTT). Based on the smoothed spectrum, we computed the percent power distribution in each frequency range, i.e., percent power in the normal range (Pow_n), bradygastric range (Pow_b), and tachygastric range (Pow_t). A more detailed description of the algorithm for the EGG analysis can be found in the Supplementary materials.

#### 2.5.2 Heart rate variability (HRV)

The raw ECG signal recordings were initially visually inspected for artifacts, which were manually removed in Acqknowledge Software before processing the digital ECG signal in MATLAB R2022a using a custom-made program. Successive R-peaks from the QRS complexes were detected in each phase (NoPain and Pain). Segments were carefully checked for missed or erroneous beats, e.g., due to arrythmias, but in our dataset none were detected. The intervals between successive R-R peaks were collected as normal-to-normal (NN) intervals. Following studies in the field (e.g., Lischke et al., 2017; Meissner, 2009; Umeda and Okifuji, 2022), we herein estimated both time-and spectral-domain parameters of the HRV, reflecting different aspects of autonomic nervous system activity [for an overview based on evidence reviewed in Shaffer and Ginsberg, 2017; Wegeberg et al., 2024, see Table 2].

**Table 2:** Overview of HRV parameters as indicators of autonomic nervous system activity.

Parameter	Autonomic nervous system activity*
SDNN	Indicates the general activity (both sympathetic and parasympathetic activity) that influences the variability in the recording period
RMSSD	Indicates parasympathetic-mediated changes
pNN50	Strongly correlated with RMSSD, therefore related to parasympathetic activity
LF	Consists of both sympathetic and parasympathetic activity; reflects baroreflex activity in resting conditions
HF	Reflects parasympathetic activity and corresponds to the heart rate variations related to the respiratory cycle.
LF/HF ratio	Estimated ratio between sympathetic and parasympathetic activity

SDNN, standard deviation of the NN intervals; RMSSD, root mean square of successive RR interval differences; pNN50, percentage of adjacent NN intervals that differ from each other by more than 50 milliseconds; LF, low-frequency component; high-frequency component; LF/HF ratio, sympathovagal balance (low frequency/high frequency ratio); * Based on evidence reviewed in Shaffer and Ginsberg (2017) and Wegeberg et al. (2024).

Regarding time-domain parameters, the ECG original signal was divided into 2-min segments as suggested by Shaffer and Ginsberg (2017) and corresponding to the segment’s length in the EGG spectral analysis. For each 2-min segment, time-domain parameters were estimated and then averaged across all segment values. The calculated parameters included the standard deviation of the NN intervals (SDNN), the root mean square of successive RR interval differences (RMSSD), and the percentage of adjacent NN intervals that differ from each other by more than 50 milliseconds (pNN50). While SDNN reflects general activity that influences the variability (i.e., both sympathetic and parasympathetic cardiac regulation, see Umetani et al., 1998; Quintana and Heathers, 2014; Shaffer and Ginsberg, 2017), both RMSSD and pNN50 are closely correlated with parasympathetic neural regulation of the heart.

For spectral-domain parameters, FFT of the HRV time series was performed and the PSD was calculated in segments of 512 samples (8 min) for the NoPain condition and 2 × 512 samples (16 min) for the Pain condition, the last resulting in an averaged spectrum. The spectral power in two frequency bands computed as output parameters in the spectral-domain HRV analysis comprised the low-frequency component (LF: 0.04–0.15 Hz), representing both sympathetic and parasympathetic regulation, the high-frequency component (HF: 0.15–0.40 Hz) representing parasympathetic regulation, and the ratio between sympathetic and parasympathetic activity (LF/HF ratio, see Shaffer and Ginsberg, 2017). A more detailed description of the algorithm for the HRV analysis can also be found in the Supplementary materials.

### 2.6 Statistical analyses

Statistical analyses focused on elucidating differences between a condition involving pain exposure (Pain condition) and a control condition without pain (NoPain condition) in the EGG and HRV measures while exploring sex differences between male and female healthy volunteers. Given that the data were not normally distributed in both conditions and for most output measures, we modelled the data using non-parametric rank-based analysis of variance ANOVA-type test for factorial longitudinal data. The statistical software packages used comprised nparLD (Noguchi et al., 2012) analyzed in R (R Core Team, 2024) and SAS® software (SAS Institute Inc, 2023). The underlying condition effects are so-called relative effects, also known as Wilcoxon–Mann–Whitney effects pX = P(X < Y), where X denotes the factor level of interest and Y denotes the fixed reference (mean) distribution. Two decisions were considered: (i) if the data under condition X tends to be smaller than those measured under condition Z or (ii) if none of the data under both conditions tend to be smaller or larger. First, a statistical model was calculated for the whole sample containing all subjects and a repeated factor *Condition* (NoPain, Pain). Then a statistical model for the same repeated factor (*Condition*) and the independent factor *Group* (females, males) and the interaction of Condition*Group was calculated. Additionally, in the statistical model of the EGG parameters, BMI was introduced as a covariate of no interest. Correlation analyses testing associations between the EGG parameters and VAS ratings for pain perception were performed by non-parametric estimation of Kendall’s Tau correlation coefficient using JASP (JASP Team, 2024).

## 3 Results

### 3.1 EGG

**Figure 2:**
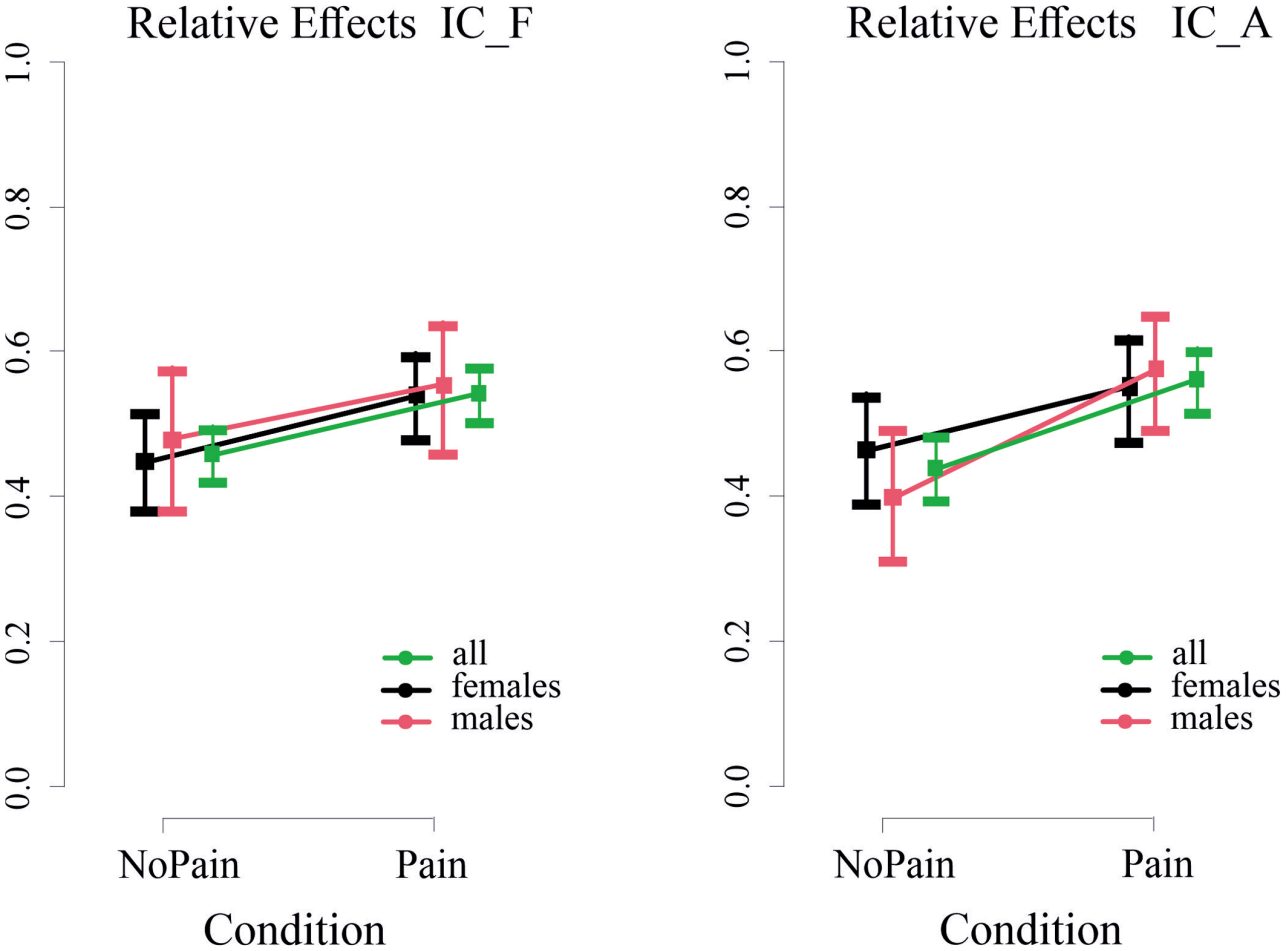
Relative *Condition* effects for the instability factor of the dominant frequency (IC_F, **left panel**) and amplitude (IC_A, **right panel**) of the EGG rhythm in the Pain vs. the NoPain conditions. The median relative *Condition* effect of the parameters (squares) and the 95% confidence intervals are presented with different colors for the whole sample of all subjects (green), females (black), and males (red). The plots were calculated by two nonparametric statistical models using the statistical toolbox nparLD (Noguchi et al., 2012) including (i) one group of all subjects and one repeated factor *Condition* and (ii) a *Group* factor (females and males), a factor *Condition* and Condition*Group interaction. Only *Condition* effects were statistically significant (for details, see text and Table 3).

Differences between the Pain and NoPain conditions were observed in five EGG parameters, comprising the IC_A, IC_F, Pow_t, Pow_n, and the NTT. Specifically, the IC_F and IC_A, reflecting the instability of the dominant EGG rhythm, were significantly higher in the Pain condition compared to the NoPain condition (see Figure 2). This was evidenced by significant effects of *Condition* (nonparametric rank-based ANOVA-type, both *p* < 0.05, see Table 3).

**Table 3:** Results of nonparametric rank-based ANOVA-type tests for EGG parameters.

Factor	IC_A	IC_F	Distribution power normal (%)	Distribution power tachygastria (%)	Distribution power bradygastria (%)	NTT	NTB
Group	*F*(1, 63.1) = 2.26 *p* = 0.1330	*F*(1, 62.5) = 0.33 *p* = 0.5664	*F*(1, 64.2) = 0.01 *p* = 0.9066	*F*(1, 61.3) = 0.05 *p* = 0.8236	*F*(1, 64.9) = 0.01 *p* = 0.9336	*F*(1, 59.9) = 0.37 *p* = 0.5406	*F*(1, 62.6) = 0.06 *p* = 0.8090
Condition	*F*(1, 67.7) = 8.99 ***p* = 0.0027**	*F*(1, 51.5) = 4.39 ***p* = 0.0362**	*F*(1, 64.4) = 5.06 ***p* = 0.0245**	*F*(1, 66.5) = 5.89 ***p* = 0.0152**	*F*(1, 66) = 3.13 *p* = 0.0770	*F*(1, 61.8) = 4.34 ***p* = 0.0371**	*F*(1, 68.7) = 0.11 *p* = 0.7424
Condition × Group	*F*(1, 67.7) = 1.05 *p* = 0.3048	*F*(1, 51.5) = 0.04 *p* = 0.8321	*F*(1, 64.4) = 0.06 *p* = 0.8124	*F*(1, 66.5) = 0.03 *p* = 0.8565	*F*(1, 66) = 0.06 *p* = 0.8033	*F*(1, 61.8) = 0.08 *p* = 0.7742	*F*(1, 68.7) = 0.26 *p* = 0.6069
BMI	*F*(1, 68.4) = 10.11 ***p* = 0.0015**	*F*(1, 68) = 7.85 ***p* = 0.0051**	*F*(1, 68.9) = 8.64 ***p* = 0.0033**	*F*(1, 67.5) = 2.94 *p* = 0.0865	*F*(1, 68.8) = 7.10 ***p* = 0.0077**	*F*(1, 67.1) = 3.73 *p* = 0.0535	*F*(1, 67.9) = 16.78 ***p* < 0.0001**

Degree of freedom were adjusted in case variances differed. IC_A, instability coefficient of the dominant amplitude; IC_F, instability coefficient of the dominant frequency; NTT, Normo-to-tachy ratio; NTB, Normo-to-brady ratio. Bold values denote statistical significance at the *p* < 0.05 level.

**Figure 3:**
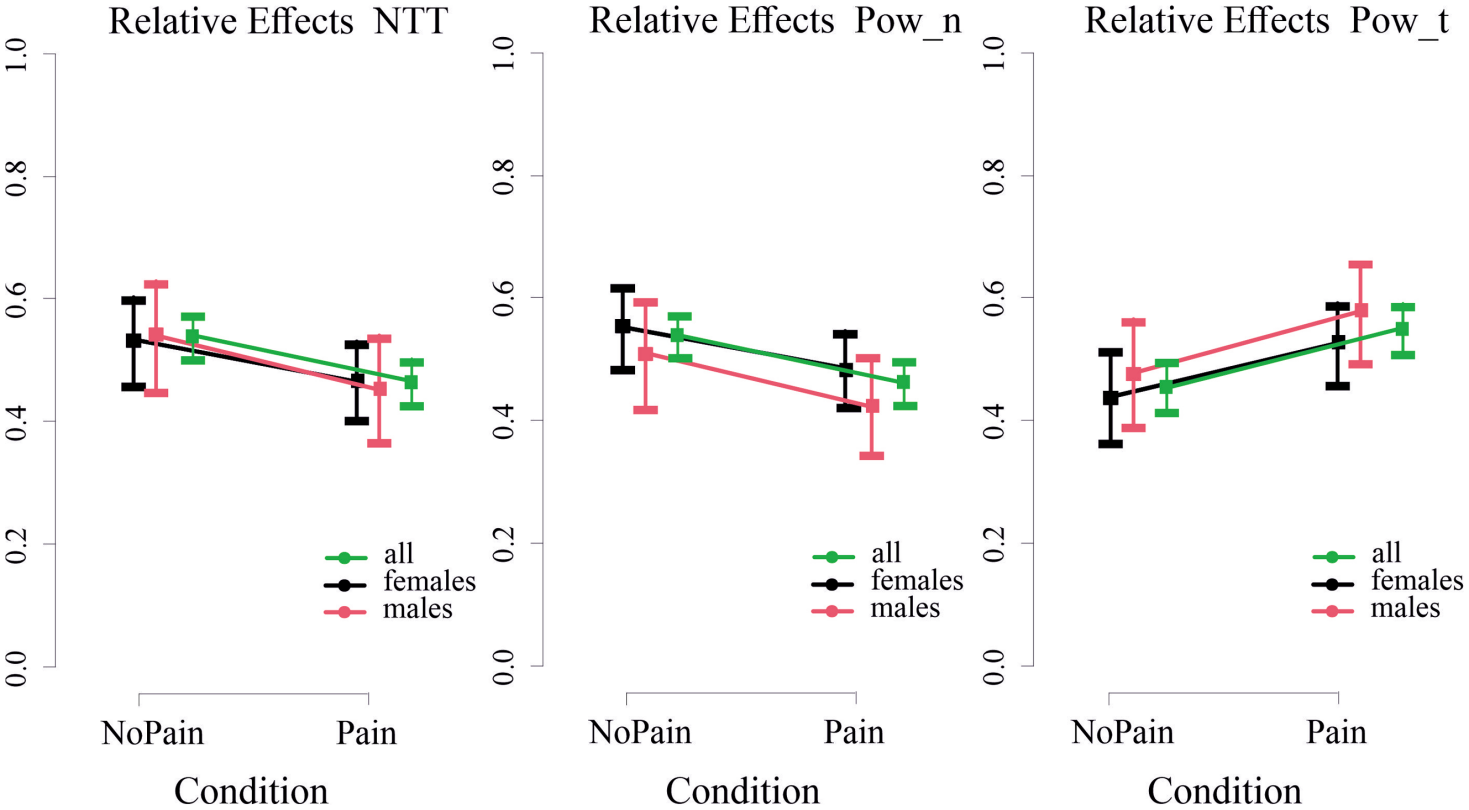
Relative *Condition* effects for the normo-to-tachy ratio (NTT, left panel) and the power distributions of normal and tachygastric power distributions (Pow_n and Pow_t; middle and right panel respectively) in the Pain vs. the NoPain conditions. The median relative *Condition* effect of the parameters (squares) and the 95% confidence intervals are presented with different colors for the whole sample of all subjects (green), females (black), and males (red). The plots were calculated by two nonparametric statistical models using the statistical toolbox nparLD (Noguchi et al., 2012) including (i) one group of all subjects and a repeated factor *Condition* and (ii) a group factor (females and males), a factor *Condition* and Condition*Group interaction. Only *Condition* effects were statistically significant (for details, see text and Table 3).

As illustrated in Figure 3, the normo-to-tachy ratio (NNT) and the power distribution of normal gastric activity (Power_n) decreased, whereas the power distribution of the tachygastric gastric activity (Power_t) increased in the Pain condition, as reflected by significant effects of *Condition* (results of nonparametric rank-based ANOVA-type statistic, all *p* < 0.05, details in Table 3). For all EGG parameter analyses, there were significant or near-significant effects of BMI as a covariate (see Table 3).

There were neither *Group* effects nor Condition*Group interaction effects for any EGG parameter, indicating that the effects of pain exposure on the EGG were not sex/gender-specific. This was further supported by the lack of sex differences in the mean dominant frequency in either phase (in the NoPain condition: females 2.82 cpm ± 0.35; males 2.89 cpm ± 0.21; in the Pain condition: females 2.79 cpm ± 0.31, males 2.79 cpm ± 0.34, all *p* > 0.05).

### 3.2 HRV

**Figure 4:**
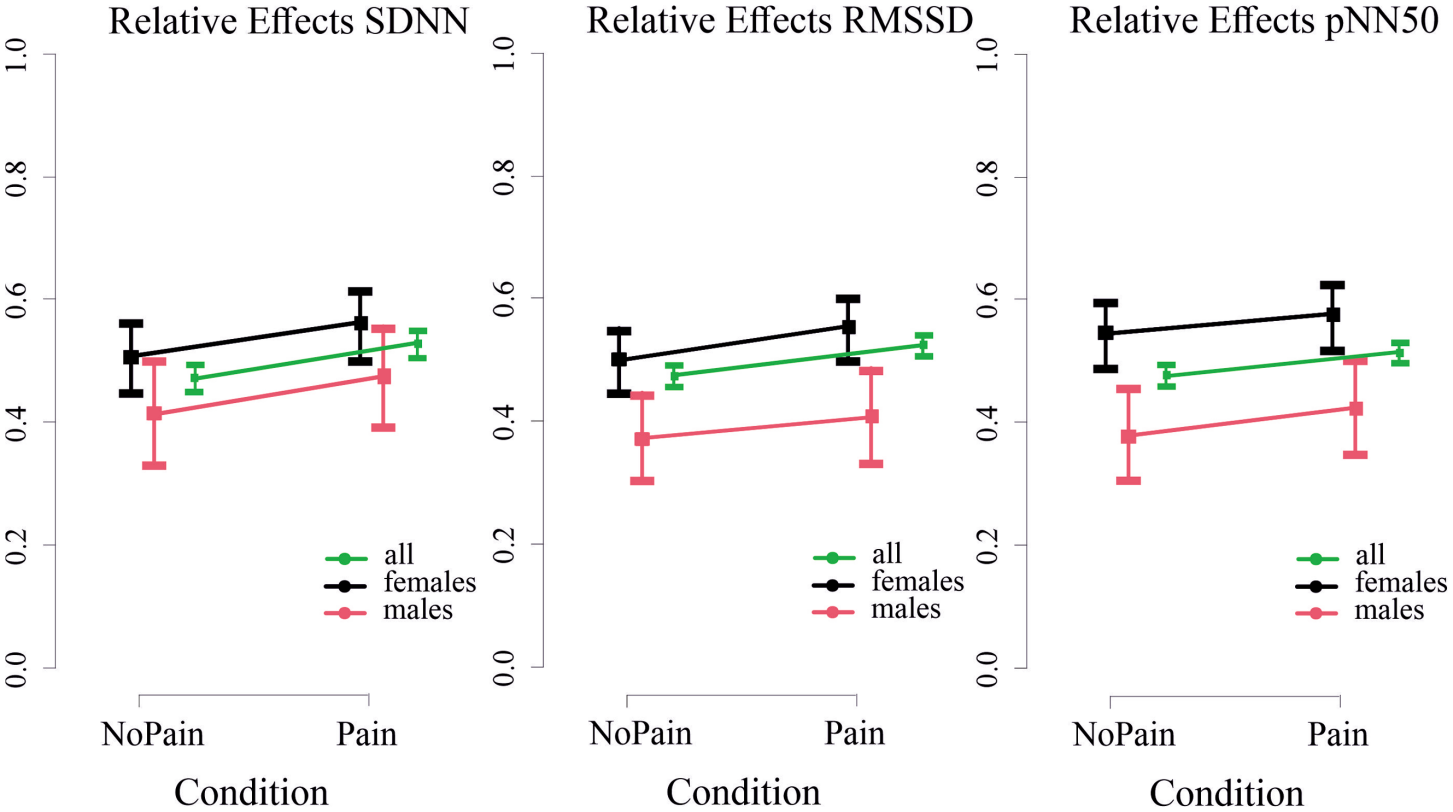
Relative *Condition* effects for the time-domain HRV parameters standard deviation of the NN intervals (SDNN, **left panel**), root mean square of successive RR interval differences (RMSSD, **middle panel**), and percentage of adjacent NN intervals that differ from each other by more than 50 milliseconds (pNN50, **right panel**) between in the Pain vs. the NoPain conditions. The median relative *Condition* effect of the parameters (squares) and the 95% confidence intervals are presented with different colors for the whole sample of all subjects (green), females (black), and males (red). The plots were calculated by two nonparametric statistical models using the statistical toolbox nparLD (Noguchi et al., 2012) including (i) one group of all subjects and one repeated factor *Condition* and (ii) a *Group* factor (females and males), a factor *Condition* and Condition*Group interaction. *Condition* effects were statistically significant for all parameters; *Group* effects were significant for RMSSD and pNN50 (for details, see text and Table 4).

For all three time-domain parameters, i.e., SDNN, RMSSD, and pNN50, we observed significant increases during the Pain condition compared to the NoPain condition, as supported by significant *Condition* effects (results of nonparametric statistics: all *p* < 0.05, see Figure 4 and Table 4). Interestingly, significant relative *Group* effects were observed for RMSSD and pNN50, with higher levels in females than males. Interaction effects of Condition*Group were not found.

**Table 4:** Results of nonparametric rank-based ANOVA-type tests for HRV parameters.

Factor	Time-domain parameters			Spectral-domain parameters		
	SDNN	RMSSD	pNN50	LF	HF	LF/HF ratio
Group	*F*(1, 63.3) = 1.93 *p* = 0.1648	*F*(1, 61.1) = 4,77 ***p* = 0.0289**	*F*(1, 61.2) = 5.97 ***p* = 0.0146**	*F*(1, 58.4) = 2.10 *p* = 0.147	*F*(1, 60.9) = 9.04 ***p* = 0.0026**	*F*(1, 57.1) = 8.14 ***p* = 0.043**
Condition	*F*(1, 56.9) = 7.57 ***p* = 0.0059**	*F*(1, 69.2) = 16.05 **p < 0.0001**	F(1, 68.8) = 9.51 ***p* = 0.0020**	*F*(1, 55.6) = 2.06 *p* = 0.1513	*F*(1, 57.3) = 0.03 *p* = 0.8557	*F*(1, 65.2) = 1.71 *p* = 0.1913
Condition × Group	F(1, 56.9) = 0.03 *p* = 0.8681	F(1, 69.2) = 0.66 *p* = 0.4181	F(1, 68.8) = 0.41 *p* = 0.5235	F(1, 55.6) = 0.56 *p* = 0.4536	F(1, 57.3) = 0.42 *p* = 0.5188	F(1, 65.2) = 0.30 *p* = 0.5816

Degree of freedom were adjusted in case variances differed. SDNN, standard deviation of the NN intervals; RMSSD, root mean square of successive RR interval differences; pNN50, percentage of adjacent NN intervals that differ from each other by more than 50 milliseconds; LF, low-frequency component; HF, high-frequency component; LF/HF ratio, sympathovagal balance (low frequency/high frequency ratio). Bold values denote statistical significance at the *p* < 0.05 level.

Regarding spectral-domain parameters, analyses revealed no significant relative effects of *Condition* for any parameter. A significant effect of *Group* was however found for the HF power and LF/HF ratio (results of nonparametric rank-based ANOVA-type statistic, both p < 0.05, details in Table 4), with higher HF power and lower LF/HF ratio in females compared to males. Interaction effects of Condition*Group were not found.

### 3.3 Correlational analyses

**Figure 5:**
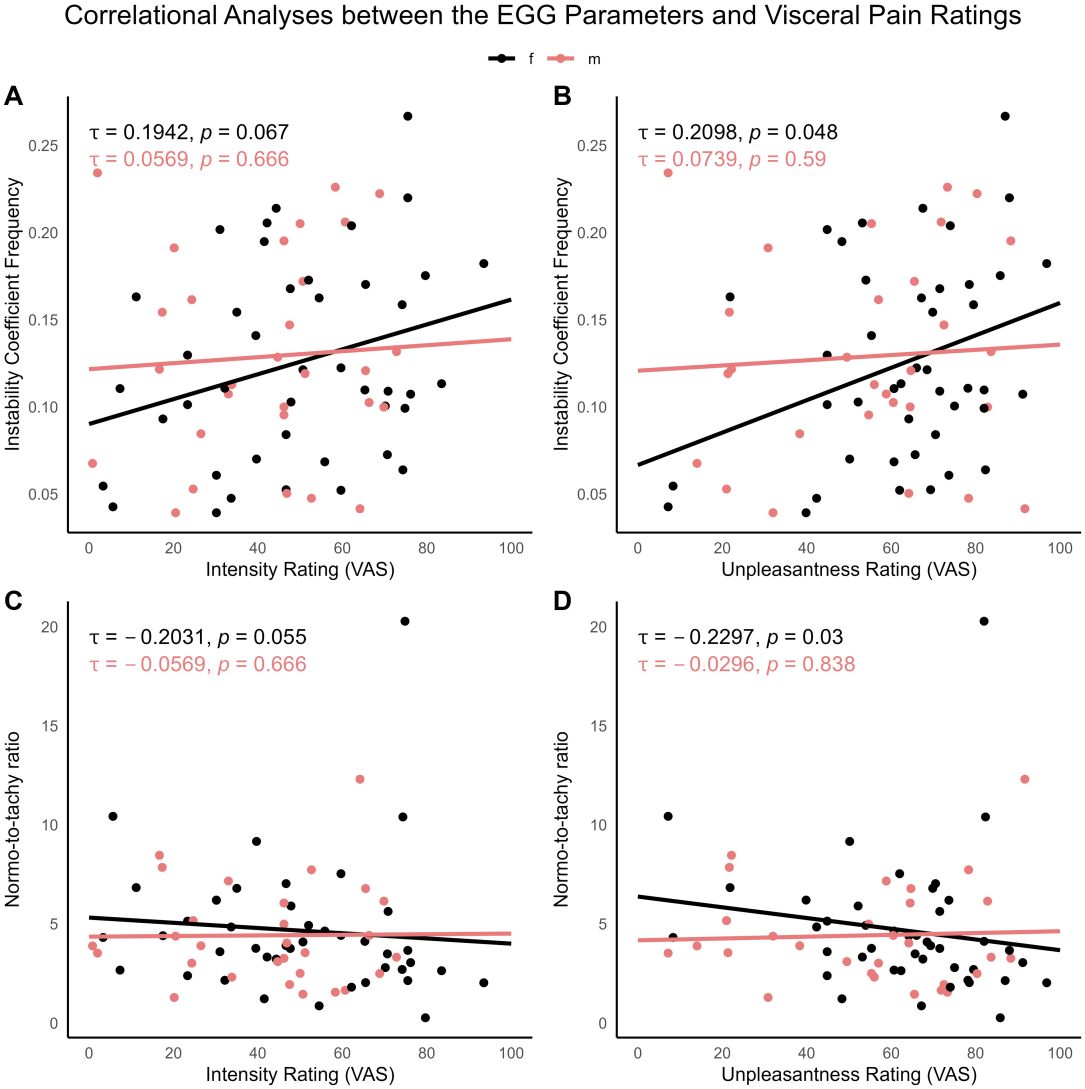
Correlations between perceived visceral pain intensity and unpleasantness, assessed with VAS, and the EGG parameters instability factor of frequency **(A,B)** and the normo-to-tachy ratio **(C,D)**. Groups are presented with different colors for females (black) and for males (red). The non-parametric estimation of Kendall’s Tau correlation coefficients was significant only in the female group (for details, see Supplementary Table S1).

For full results of non-parametric correlational analyses between visceral pain perception and psychophysiological measures that showed reactivity to pain, see Supplementary Table S1. Interestingly, perceived visceral pain unpleasantness correlated with the instability factor frequency, as well as with the normo-to-tachy ratio in the female but not the male group (Figure 5 and Supplementary Table S1). Somatic pain perception, on the other hand, did not correlate with any psychophysiological measure (data not shown).

## 4 Discussion

This study investigated whether EGG and HRV can serve as psychophysiological markers in experimental pain research involving the gut-brain axis. We analyzed EGG and ECG data collected during baseline and pain phases, examining responses to a series of visceral (rectal distension) and somatic (cutaneous heat) pain stimuli. Additionally, we investigated sex differences in EGG and HRV responses, providing insight into potential sex-specific mechanisms relevant to underlying pain processing and the autonomic regulation of the microbiota-brain-gut axis.

### 4.1 Electrogastrography

In this first human study examining the impact of acute pain on EGG measures, we observed significantly increased instability in gastric slow wave frequency and amplitude during pain exposure. Additionally, there were changes in the power distribution of the slow wave, comprising increased tachygastria and a reduced normo-to-tachy ratio, paralleled by a decrease in normogastric spectral power. These findings indicate that acute pain alters gastric myoelectrical activity, potentially impairing gastric motility and delaying gastric emptying. Similar pain-induced gastric dysrhythmias with an uncoupling of gastric slow waves in temporal and spatial regularity, respectively, have indeed been linked to delayed gastric emptying in functional dyspepsia (Riezzo et al., 2013), and comparable disruptions in gastric and jejunal myoelectrical activity have been observed in dogs exposed to painful rectal distension (Abo et al., 2000). Our results suggest that acute pain experienced in the lower abdominal region could negatively affect gastric motility, predisposing individuals to upper abdominal symptoms like nausea, vomiting, early satiety, or postprandial fullness, which are common in functional dyspepsia and eating disorders.

We found no significant sex differences in EGG responses to pain. While the uneven sample (29 males, 43 females) is a limitation that warrants cautious interpretation, our robust non-parametric analysis and the overall sample size are comparable to or exceed similar studies in the field. This lack of sex differences aligns with previous research on visceral sensitivity assessed by rectal distension not only in healthy subjects (Icenhour et al., 2021; Horing et al., 2013) but also in IBS patients (Roberts et al., 2023), and other EGG studies reporting no sex effects (Weimer et al., 2018). Nonetheless, EGG responses in different clinically relevant experimental contexts, such as nausea paradigms, may show more pronounced effects in women, as suggested by evidence that decreases in normo-to-tachygastric ratios were observable only in females during nausea (Meissner et al., 2020). Postprandial EGG changes are also reportedly influenced by sex and menstrual cycle (Parkman et al., 1996), the latter possibly introducing variability that we were unable to consider herein as this would have required a much larger sample size.

Although our pain paradigm, combining visceral and somatic stimuli, did not allow us to isolate EGG changes by pain modality, correlational analyses offered valuable insights. We found that pain-induced gastric dysrhythmia (i.e., instability in slow wave frequency and normo-to-tachy ratio) was associated with the unpleasantness of visceral, but not somatic, pain. This suggests that visceral pain is more strongly linked to gastric slow-wave instability and increased tachygastric activity. Moreover, higher visceral pain unpleasantness was correlated with greater EGG alterations, but only in female participants. In the absence of overall sex differences, these results indicate that sex-specific factors may influence pain perception or reporting, potentially affecting psychophysiological responses regulated by the autonomic nervous system. Given previous findings that placebo effects on gastric myoelectric activity were exclusive to females (Meissner et al., 2020), and that expectations can modulate EGG measures in placebo studies (Meissner, 2009), it is possible that psychosocial factors, such as expectations, fear, and stress, interact differently along the gut-brain axis based on sex and/or gender variables, especially in patients. This aligns with evidence from neurogastroenterology, where sex differences in response to such factors have been observed (e.g., Benson et al., 2019; Labrenz et al., 2020), as recently reviewed in Labrenz et al. (2023). This idea is further supported by the well-established role of female sex as a vulnerability factor for abdominal pain-related disorders of gut-brain interaction like IBS (Zia et al., 2022) and other chronic pain conditions (Mills et al., 2019).

### 4.2 HRV

Previous experimental pain studies have used HRV analyses to assess autonomic mechanisms involved in pain perception and modulation, particularly with heat pain (e.g., Nahman-Averbuch et al., 2016b; Nahman-Averbuch et al., 2016a) and visceral pain (e.g., Rubio et al., 2015). HRV has also been explored for its potential role in understanding autonomic mechanisms underlying pain treatment effects (e.g., Gholamrezaei et al., 2021), and has proved useful to elucidate a decoupling of the ANS and the nociceptive system as a possible mechanism underlying abnormal visceral perception in IBS (Kano et al., 2019). While HRV has been incorporated in some EGG studies (Meissner et al., 2009; Witt et al., 2012; Levanon et al., 2002; Minagawa et al., 2013), it has never been combined with EGG in the context of clinically relevant pain exposure. Our HRV analyses revealed significant increases in the time-domain parameters SDNN, RMSSD, and pNN50 during exposure to pain, whereas the spectral-domain parameters HF component, LF component, and LF/HF ratio did not differ from the baseline condition. Considering that SDNN represents not only the sympathetic branch of the ANS but also partly the parasympathetic activation, the increase of SDNN might reflect a change in sympathovagal regulation, paralleled by greater pain-induced instability of the gastric slow wave and tachygastric activity. Other studies reported an increase in SDNN and RMSSD induced by pain induced by ice water immersion (Konstantinou et al., 2020) or by trigeminal capsaicin stimulation (Poulsen et al., 2019). Similarly, in research involving thermal pain, a positive correlation between RMSSD and higher pain ratings in highly anxious subjects was observed (Nahman-Averbuch et al., 2016b), and the same group also reported higher RMSSD in female group compared to males (Nahman-Averbuch et al., 2016a). Consistent with this finding, we also observed significant differences between male and female groups in several time-and spectral-domain HRV measures. However, only time-domain parameters were sensitive to pain. Hence, some sex differences in HRV measures appear to exist independent of autonomic reactivity to pain. These differences also include spectral-domain parameters which are consistent with the resting HRV findings from Min et al. (2023, 2024). They found a greater HF power in females compare to males, indicating a relative dominance of parasympathetic activity in females (Koenig and Thayer, 2016). It is intriguing to speculate that sex differences in autonomic regulation, even if seemingly unrelated to pain in healthy samples, may play a role in explaining the female preponderance of chronic visceral pain or in other symptoms arising from perturbations of gut-brain axis integrity in patients. Given the lack of sex differences in the modulation of the EGG by pain, it is important to consider prior evidence suggesting that gastric motility can be modulated independently from changes in general autonomic activity in healthy subjects (Meissner, 2009). Hence, while both EGG and HRV can be used in combination, it appears that measures may provide distinct findings that do not necessarily align. Our results are reflective of the complexity of the coordination between GI tract regulation and cardiac autonomic function. Novel ambulatory recording devices, such as Cervical Electroneurography (Bu et al., 2022), may prove useful developments to non-invasively assess changes in autonomic nervous system activity induced by pain or other stressors, and should be explored in future studies as a possible complementary or alternative measure to ECG-based HRV analyses.

## 5 Conclusion and future directions

In summary, this study demonstrates that EGG and HRV are sensitive psychophysiological tools for assessing acute pain in healthy volunteers that are useful for the investigation of pain mechanisms along the gut-brain axis, with implications for research into numerous clinical conditions involving the gut-brain axis ranging from abdominal pain and visceral hypersensitivity (e.g., Farmer and Aziz, 2013), such as in IBS (Kano et al., 2019), to upper digestive tract organic conditions with complex ANS dysregulation as part of the pathogenesis (e.g., Budzyński and Kłopocka, 2014). However, our findings should be interpreted with caution, especially regarding sex differences, and considering the unequal durations of the baseline and pain phases, which is a limitation. Future studies should recruit larger and more sex−/gender balanced samples of volunteers, ideally incorporating a larger age range for enhanced knowledge regarding diversity aspects and in order to elucidate factors relevant to interindividual differences not only in healthy volunteers but ideally in patients or at-risk populations with vulnerability factors. Another critical point is the fact that we did not measure respiration, and hence cannot with certainty exclude or control for possible pain-related respiratory changes on SDNN, RMSSD, and pNN50. However, our pain stimuli were not only cued by visual signals, avoiding a sudden or unexpected pain experience. They were also of relatively long duration with slow inflation speed of the rectal balloon (and hence the matched slow thermode temperature rise), effectively avoiding any sharp or sudden severe pain experiences that would more likely elicit respiratory changes or movement (the latter specifically analyzed in one of our earlier research using a similar pain paradigm, finding no evidence of pain-related movement changes; Koenen et al., 2021). Nevertheless, future work should ideally consider respiratory activity. An additional critical consideration includes relatively small effects, especially for LF/HF ratio and NTT. Despite these limitations, our study is the first to show that acute lower abdominal pain affects EGG measures linked to gastric motility and emptying, which has clinical relevance and calls for future research. These findings suggest a potential mechanism underlying the multiple symptoms experienced by patients with disorders of gut-brain interaction. This aligns with evidence that abnormal electrogastrograms in IBS occur mainly when dyspepsia is present (Leahy et al., 1999). Additionally, our data indicate that exposure to pain may increase the risk of gastric symptoms, particularly in individuals with other risk factors, such as female sex/gender or in patients with high levels of stress or pain-related fear that may exacerbate the impact of pain. Together, our findings call for more experimental and clinical pain research applying EGG and HRV as tools in patients with chronic visceral pain.

## Data Availability

The datasets presented in this study can be found in online repositories. The names of the repository/repositories and accession number(s) can be found at: OSF (https://osf.io/awr6k/).
