# Supplementary material for: Sex differences in the reactivity of gastric myoelectrical activity and heart rate variability as putative psychophysiological markers in human pain research
Source: Front Neurosci. 2024 Dec 11;18:1502752. doi: 10.3389/fnins.2024.1502752 (PMC11670328; doi:10.3389/fnins.2024.1502752)
Supplement: Supplementary file 1 [file Data_Sheet_1.docx]

***Supplementary Material***

# Supplemental Methods

# S1: Algorithm of the EGG signal analysis

Before starting the analysis, the EGG signal was interpolated by an increment of 128 samples based on the ratio between the acquisition sample rate (2000 Hz) set by Biopac Software and the custom setting of the signal waveform sample rate of the EGG signal (15.625 Hz). Then, the EGG signal spectra were estimated by Fourier transform. The recording time in both conditions suggested the non-stationarity of the signal. The Fourier transform is correctly calculated if the Nyquist theorem is fulfilled, and the signal is considered stationary. These requirements depend on sampling frequency and the length of the signal, which demands an optimal signal length of 2 min. Hence, the recording trials were divided into 2-min segments with an overlap of 1-min, resulting in N-1 number of segments, where N is the trial length in minutes. On each 2-min segment, the Fast Fourier Transform (FFT) was applied, and the running spectra (RS) were estimated. Corresponding to the sampling frequency, each 2-min segment contains 1876 samples. In FFT computations the signal length in samples should be a power of 2 (256, 512, 1024, 2048…). Therefore, for a more precise calculation of the RS, the sample number of each 2-min segment (1876 samples) and 1-min overlap (938 samples) were rounded to 2048 to 1024 samples, respectively. For the calculation of the RS, the *“periodogram”* function in MATLAB was used, which estimates the power spectral density (PSD) of a stationary random process, and the Hamming window was applied to smooth the signal’s ends, reducing the spectral leakage. Figure S1 illustrated the spectral analysis of the EGG signal.


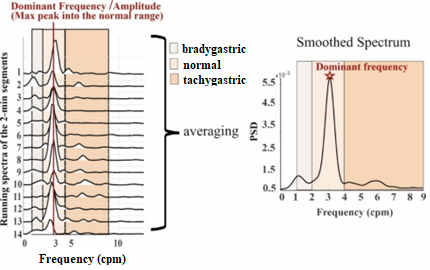


**Supplemental Figure 1 (Fig. S1).** Example based on data from one participant. The left figure shows a waterfall diagram of the calculated running spectra from individual data, while the right figure shows the smoothed spectrum resulted from averaging all the running spectra across one two-minute segment. Three different frequency ranges (normal, tachy- and brady-gastric activity) are marked with different background color shades.

The highest peak into the normal range representing the slow-wave gastric rhythm around 3 cycle per minute (cpm) is denoted as dominant frequency or dominant amplitude (DF and DA, respectively). According to EGG studies in the field (Chen et al., 1999; Parkman et al., 2003; Meissner, 2009; Yin and Chen, 2013), the signal was described by several quantitative parameters. Based on the running spectra, the deviation in amplitude and frequency (i.e. instability coefficient, IC, in amplitude or frequency) through all RS were calculated by the formula:

${IC= SD}/\mathrm{MEAN}$,

where MEAN is the mean between the accumulated DA or DF across all running spectra and SD is the standard deviation between them. Except for the normal frequency band, the EGG spectrum contains power into two frequency bands containing abnormal activity. The frequency range from 2 – 4 cpm represent the normal activity, while the ranges from 1.2 – 2 cpm and 4 – 9 cpm represent the abnormal bradi-gastric and tachi-gastric activity, respectively (Fig. 1). Furthermore, the normal-to-tachi ratio (NTT) was calculated, for each running spectrum as follows (Meissner et al., 2020): *PSD* into frequency range 2 cpm – 4 cpm (normal) for each 2-min segment was divided by the *PSD* into the range 4 cpm – 9 cpm (tachi):

$\mathrm{NTT}=\sum_{i=1}^{n} \frac{PSD(normal)}{PSD(tachi)}$*,*

where NTT represents the mean of the NTTs calculated for each segment, n – number of segments. Another three parameters were calculated based on the smoothed spectrum, the average across all running spectra (Fig. 1, right). The percent of power distribution for each power frequency band was calculated according to formula:

$PSD\left( range distribution \% \right)=\frac{PSD(range)}{PSD\left( total range \right)\times100}$,

where PSD distribution is the percent of the Power into a certain frequency range (normal, tachi, bradi) divided by the total Power into a frequency range (1.2 cpm – 9 cpm). An illustration of the processed result on one participant is shown in Figure S2.


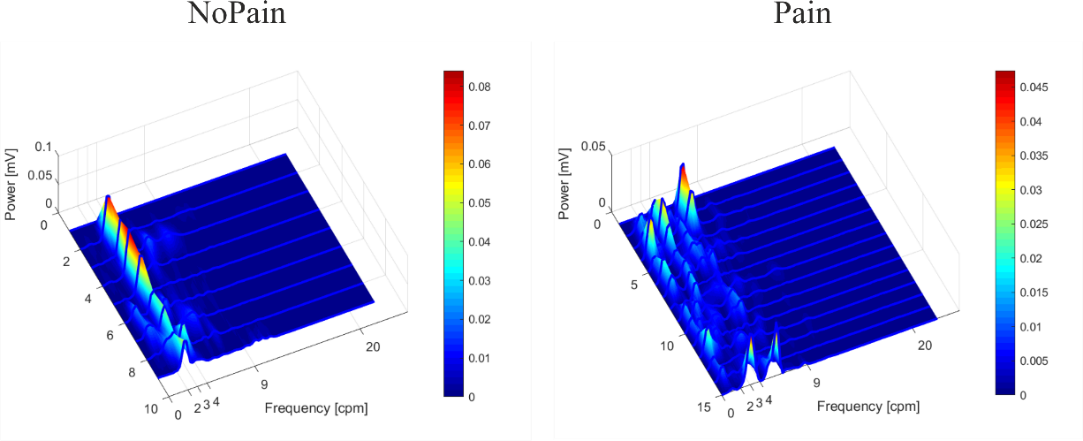


**Supplemental Figure 2 (Fig. S2).** Illustration of individual 3-D running spectra from the NoPain (left) and Pain (right) conditions.

# S2: Algorithm of the HRV analysis from the ECG signal

The ECG signal was digitized with a sampling rate of 2000 Hz. The raw digital signal was inspected visually for artifacts before further processing in MATLAB R2022a using a custom-made script. Initially, in the digitalized ECG signal to each phase (NoPain and Pain), successive QRS complexes were detected. Segments were carefully checked for missed or erroneous beats, e.g. due to arrythmias, but in our dataset none were detected. Because of the low-frequency fluctuation of ECG, the peak-to-peak detection method used by Biopac Software did not give reliable results. Therefore, we used the ‘Pan-Tomkins’ algorithm implemented by MATLAB (Pan and Tompkins, 1985). The intervals between successive R-R peaks are collected in milliseconds units and resulting in a HRV curve. Then, both the time and frequency domain parameters of the HRV were estimated.

## *Time-domain estimations*

The ECG original signal was divided into 2-minute segments as suggested by Shaffer and Ginsberg (2017) and corresponding to the segment’s length in the EGG spectral analysis. The time domain parameters calculated in this study are as follows:

1. Standard deviation of NN intervals (SDNN):

$$SDNN=\sqrt{\frac{1}{N-1}\sum_{i=1}^{N} [{{NN}_{i}-mean NN)]}^{2}}$$

Both sympathetic and parasympathetic nervous system contribute to SDNN (Umetani et al., 1998).

1. Root mean square of successive RR interval differences (RMSSD):

$$RMSSD=\sqrt{\frac{1}{N-1}\sum_{i=1}^{N} ({{NN}_{i+1}-{NN}_{i})}^{2}}$$

RMSSD is widely acknowledged as a measure of cardiac parasympathetic activity (Shaffer and Ginsberg, 2017) and is highly correlated with the high frequency component in the frequency domain (see Spectral-domain estimation section below, and Kleiger et al., 2005).

1. Percentage of adjacent NN intervals that differ from each other by more than 50 milliseconds (pNN50):

$$pNN50=\frac{NN50}{N-1} \times100\%$$

Like RMSSD, pNN50 closely correlated with parasympathetic activity (Umetani et al., 1998).

Theses parameters are estimated for each 2-min segment and then averaged across all segments.

## *Spectral-domain estimation*

The Fourier PSD of the HRV time series was calculated using Welch’s method (function *“pwelch”* in MATLAB). Thus, the HRV curve was resampled by 1 Hz (∆t = 1s), which means, the highest component in the spectrum that can be detected in the spectrum is f = ½ ∆t (0.5 Hz, see Kuusela, 2016). Welch’s function also offers the option to calculate the PSD of segments and to average them thereafter. Additionally, a Hanning window was applied to each segment to reduce the spectral leakage. The duration of both signals in conditions (NoPain and Pain) was 10 min and 16 min, respectively. The PSD spectrums of the HRV curves were computed in segments of 512 samples (8 min) for the NoPain condition and 2 × 512 samples (16 min) for the Pain condition, the last resulting in an averaged spectrum. We used three spectral domain parameters in this study, i.e., the low-frequency component (LF: 0.04 – 0.15 Hz), the high-frequency component (HF: 0.15 – 0.4 Hz) and the sympathovagal balance ratio (LF/HF Ratio). Figure S3 shows the plot required for visual inspection. The plot illustrates the R-peak detection markers on the ECG signals, HRV curve, and PSD spectra of the HRV in both NoPain and Pain conditions.


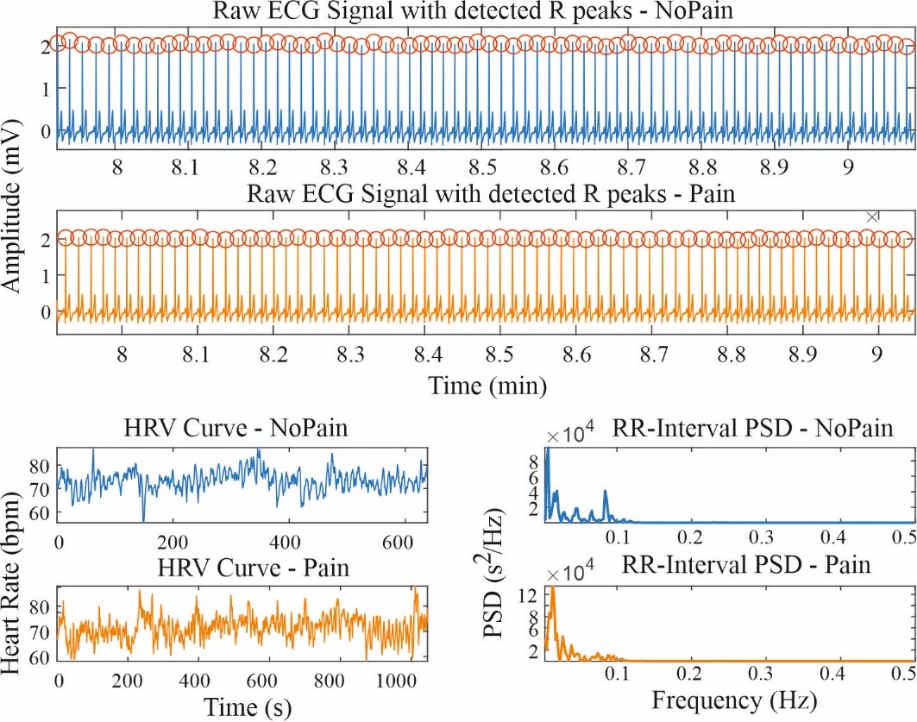


**Supplemental Figure 3 (Fig. S3).** Visual inspection of the HRV algorithm for real individual data in MATLAB.

# Supplemental Table S1. Results of non-parametric correlational analyses between visceral pain perception and psychophysiological measures

| **Group** | **Visceral pain intensity**  **vs**  **IC_F** | **Visceral pain intensity**  **vs**  **NTT** | **Visceral pain unpleasantness**  **vs**  **IC_F** | **Visceral pain unpleasantness**  **vs**  **NTT** | **Visceral pain intensity**  **vs**  **RMSSD** | **Visceral pain intensity**  **vs**  **pNN50** | **Visceral pain unpleasantness**  **vs**  **RMSSD** | **Visceral pain unpleasantness**  **vs**  **pNN50** |
| --- | --- | --- | --- | --- | --- | --- | --- | --- |
| Females | τ = 0.194  p = 0.067 | τ = - 0.203  p = 0.055 | τ = 0.210  **p = 0.048** | τ = - 0.230  **p = 0.030** | τ = 0.046  p = 0.668 | τ = 0.048  p = 0.653 | τ = 0.068  p = 0.523 | τ = 0.039  p = 0.714 |
| Males | τ = 0.057  p = 0.666 | τ = - 0.057  p = 0.666 | τ = 0.074  p = 0.574 | τ = - 0.030  p = 0.822 | τ = - 0.096  p = 0.464 | τ = - 0.037  p = 0.778 | τ = - 0.005  p = 0.970 | τ = - 0.025  p = 0.851 |
| Whole sample | τ = 0.121  p = 0.134 | τ = - 0.128  p = 0.112 | τ = 0.136  p = 0.092 | τ = - 0.137  p = 0.089 | τ = 0.049  p = 0.543 | τ = 0.069  p = 0.389 | τ = 0.069  p = 0.392 | τ = 0.052  p = 0.515 |

τ : Kendall’s Tau Coefficient; IC_F: Instability Coefficient of the Dominant Frequency; NTT: Normo-to-tachy ratio; Perceived visceral pain intensity and unpleasantness were assessed using VAS; RMSSD: root mean square of successive RR interval differences; pNN50: percentage of adjacent NN intervals that differ from each other by more than 50 milliseconds.

# Supplemental References

Chen, J. D. Z., Zou, X., Lin, X., Ouyang, S., and Liang, J. (1999). Detection of gastric slow wave propagation from the cutaneous electrogastrogram. *Am J Physiol Gastrointest Liver Physiol* 277, 424–430. doi: 10.1152/ajpgi.1999.277.2.g424

Kleiger, R. E., Stein, P. K., and Bigger, J. T. (2005). Heart rate variability: Measurement and clinical utility. *Annals of Noninvasive Electrocardiology* 10, 88–101. doi: 10.1111/j.1542-474X.2005.10101.x

Kuusela, T. (2016). *Heart Rate Variability (HRV) Signal Analysis*., eds. M. V. Kamath, M. Watanabe, and A. Upton. Boca Raton: CRC Press. doi: 10.1201/b12756

Meissner, K. (2009). Effects of placebo interventions on gastric motility and general autonomic activity. *J Psychosom Res* 66, 391–398. doi: 10.1016/j.jpsychores.2008.09.004

Meissner, K., Lutter, D., von Toerne, C., Haile, A., Woods, S. C., Hoffmann, V., et al. (2020). Molecular classification of the placebo effect in nausea. *PLoS One* 15. doi: 10.1371/journal.pone.0238533

Pan, J., and Tompkins, W. J. (1985). A Real-Time QRS Detection Algorithm. *IEEE Trans Biomed Eng* BME-32, 230–236. doi: 10.1109/TBME.1985.325532

Parkman, H. P., Hasler, W. L., Barnett, J. L., and Eaker, E. Y. (2003). Electrogastrography: A document prepared by the gastric section of the American Motility Society Clinical GI Motility Testing Task Force. *Neurogastroenterology and Motility* 15, 89–102. doi: 10.1046/j.1365-2982.2003.00396.x

Shaffer, F., and Ginsberg, J. P. (2017). An Overview of Heart Rate Variability Metrics and Norms. *Front Public Health* 5, 1–17. doi: 10.3389/fpubh.2017.00258

Umetani, K., Singer, D. H., McCraty, R., and Atkinson, M. (1998). Twenty-Four Hour Time Domain Heart Rate Variability and Heart Rate: Relations to Age and Gender Over Nine Decades. *J Am Coll Cardiol* 31, 593–601. doi: 10.1016/S0735-1097(97)00554-8

Yin, J., and Chen, J. D. Z. (2013). Electrogastrography: Methodology, validation and applications. *J Neurogastroenterol Motil* 19, 5–17. doi: 10.5056/jnm.2013.19.1.5
